# Multi-omics integration with weighted affinity and self-diffusion applied for cancer subtypes identification

**DOI:** 10.1186/s12967-024-04864-x

**Published:** 2024-01-19

**Authors:** Xin Duan, Xinnan Ding, Zhuanzhe Zhao

**Affiliations:** 1 School of Artificial Intelligence, Anhui Polytechnic University, Wuhu, 241000 China; 2 School of Biomedical Engineering, Shenzhen Campus of Sun Yat-Sen University, Shenzhen, 518000 China; 3 College of Electrical Engineering, Anhui Polytechnic University, Wuhu, 241000 China; 4 Center for Robot Performance Testing and reliability assessment, Anhui Polytechnic University, Wuhu, 241000 China; 5 Anhui Provincial Key Laboratory of Discipline Co-Construction On Intelligent Equipment Quality and Reliability, Wuhu, 241000 China

**Keywords:** Cancer heterogeneity, Muti-omics, Weighted affinity, Self-diffusion

## Abstract

**Background:**

Characterizing cancer molecular subtypes is crucial for improving prognosis and individualized treatment. Integrative analysis of multi-omics data has become an important approach for disease subtyping, yielding better understanding of the complex biology. Current multi-omics integration tools and methods for cancer subtyping often suffer challenges of high computational efficiency as well as the problem of weight assignment on data types.

**Results:**

Here, we present an efficient multi-omics integration via weighted affinity and self-diffusion (MOSD) to dissect cancer heterogeneity. MOSD first construct local scaling affinity on each data type and then integrate all affinities by weighted linear combination, followed by the self-diffusion to further improve the patients’ similarities for the downstream clustering analysis. To demonstrate the effectiveness and usefulness for cancer subtyping, we apply MOSD across ten cancer types with three measurements (Gene expression, DNA methylation, miRNA).

**Conclusions:**

Our approach exhibits more significant differences in patient survival and computationally efficient benchmarking against several state-of-art integration methods and the identified molecular subtypes reveal strongly biological interpretability. The code as well as its implementation are available in GitHub: https://github.com/DXCODEE/MOSD.

**Supplementary Information:**

The online version contains supplementary material available at 10.1186/s12967-024-04864-x.

## Introduction

With the advent of high-throughput sequencing technology, which can sequence thousands of genes in a short period of time, it is easier to obtain molecular data at different levels, such as transcriptome, genome, metabolome, and epigenome. Analysis of multi-omics data can provide the complement information of molecular characteristics in each data level and hence can derive more useful insights into the complex biological processes. Moreover, integrating approaches contribute to evaluate the flow of information among omics levels and narrow the gap from genotype to phenotype. The merits of integrating multi-omics data over single omics have been proved in various studies [1, 2] and the availability of multi-omics data has attracted much more interest in the field of biology and bioinformatics, especially in cancer treatment. Most cancer types are not single diseases but rather contains different molecular subtypes. Those molecular subtypes underline diverse clinical characterizations which are closely related to the treatment response [3]. The precise understanding of cancer heterogeneity is a requirement for efficient targeted treatment and precision medicine. Many strategies for identifying cancer molecular subtypes often use gene expression profiling to analyze the heterogeneity of tumors [4, 5]. However, tumor heterogeneity also exists at other molecular data types, such as microRNA and DNA methylation.

In cancer subtyping, applying multi-omics data along with clinical information to predict patient outcome has taken front seat in personalizing therapy and understanding cancer mechanism. In recent decades, various multi-omics integration methods for cancer subtyping have emerged, of which can be mainly grouped into two classes based on whether the method uses networks or Bayesian to model the integration. The network-based methods take either advantage of the connectivity from all data types or employ similarity networks constructed from samples correlation analysis. The popular network-based approach is similarity network fusion (SNF) [6] which integrates multi-omics data via a nonlinear network combination. SNF first create an individual similarity graph for each data type and then fuse all graphs into one final network by message-passing theory. Neighborhood based multi-omics clustering (NEMO) [7] is another network -based integration method that is inspired and builds on SNF and rMKL-LPP [8]. NEMO captures the local neighborhoods of samples in each omic and then calculates the final graph by averaging the relative similarity matrices. The advantage of NEMO is that it is applicable on partial data set. Ramazzotti et al. [9] proposed a network-based cancer data integration method called CIMIR (Cancer Integration via multi-kernel learning). CIMIR first construct Gaussian kernel functions with 55 different parameters for each data type and then optimizes all the Gaussian kernel functions into an similarity graph, followed by performing K-means on the integrated graph to identify cancer molecular subtypes. PINSPlus [10] observes the small changes in quantitative assays to estimate how often each pair of samples is grouped together. Spectrum [11] is a fast and density-aware spectral method for single and multi-omics data clustering.

Bayesian approaches assume that the prior probability distribution fits a specific model relying on one or more parameters. Shen et al. [12]. Developed a joint variable model named iCluster for integrative clustering which simultaneously infer multiple data types to generate a single cluster assignment for samples. LARcluster [13] incorporates an integrative probabilistic model with low-rank approximation to find the shared principal low-dimension subspace for classification of omics data. PARADIGM [14] infers the activities of patient-specific biological pathways by Bayesian factor graphs to combine multiple omics-scale measurements on a single sample.

A drawback common to the current integration approaches is that they have relatively poor computational efficiency due to the necessity to infer numerous petameters. Moreover, the weight coefficient in every data type is treated equally, which may be not biologically appropriate for the multi-omics datasets with large differences in feature size.

**Fig. 1:**
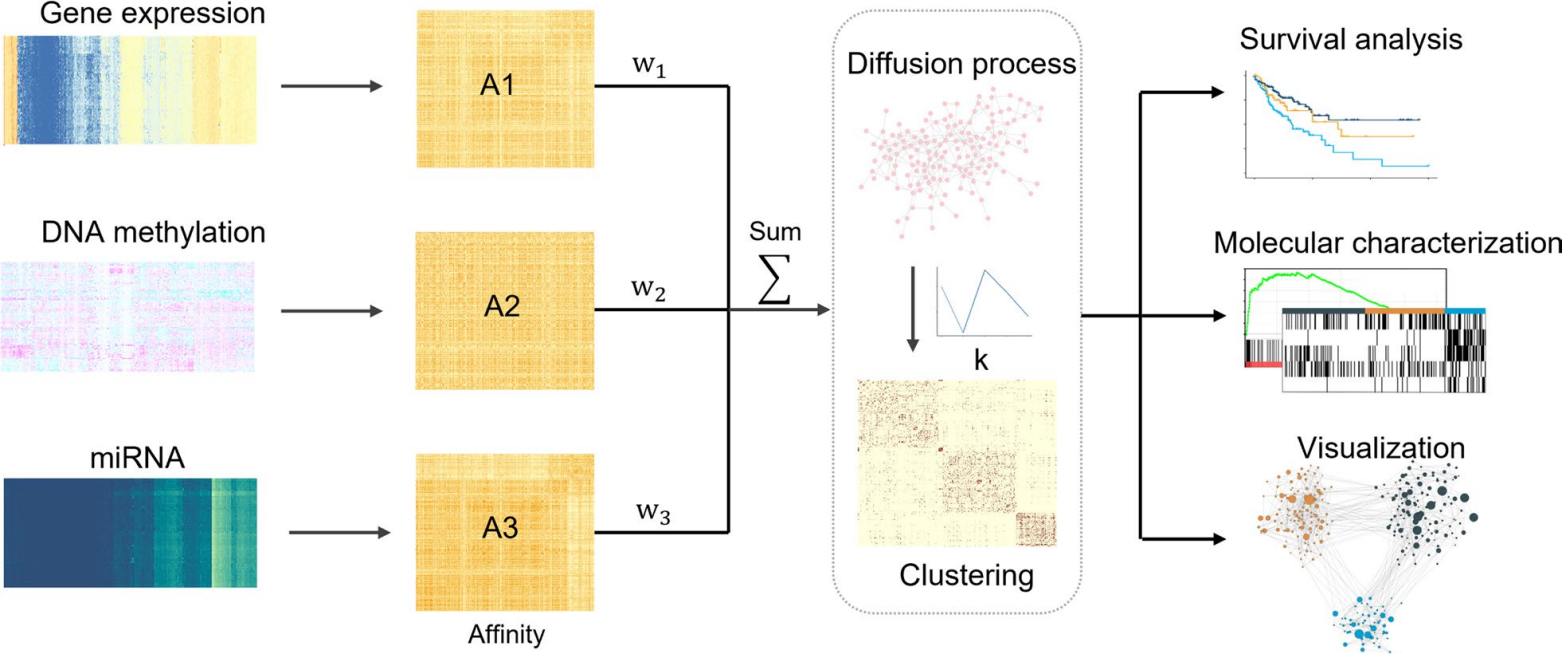
Schematic workflow of MOSD**.** When integrating multi-omics datasets, MOSD first create the affinity using each data type where rows are patients and columns are gene features. MOSD then assign weights for the affinities and perform linear combination in integration step, followed by implementing self-diffusion to enhance the similarity of integrated network. Spectral clustering is used to obtain the patients labels with the optimal clustering number estimated by separation cost method. The identified subtypes are evaluated by survival analysis and biological analysis

To overcome these drawbacks, we proposed a fast multi-omics integration approach via weighted affinity and self-diffusion (MOSD) for cancer subtyping (Fig. 1). MOSD first create an affinity for each data type using local scaling method which infers the self-tuning of patient-to-patient distances to eliminate scales differences. Different weights are then assigned for each data type instead of using equal weight importance. We measure the weight of each data type by exponentiating the ratio of features size based on the assumption that larger features contribute more information to the final integrated network. The integrated network can be obtained via the summation of the affinities multiplying by weights coefficient. MOSD can thus offer us insight into which data types are most informative in the integration for cancer subtyping. Self-diffusion process is finally performed on the integrated network to enhance the network similarity. Self-diffusion belongs to diffusion-based metric learning approaches and is related to diffusion map [15, 16]. Self-diffusion assumes that long-range similarities can be estimated by accumulation of local similarities, therefore facilitating the with-cluster similarity of samples. After obtaining the integrated network, we applied spectral clustering to assign labels to patients since spectral clustering is superior in capturing global structure of a graph. Moreover, we provide a method to estimate the optimal clustering number based on the diffused network. The advantage of our MOSD approach is that it uses weighted local scaling affinities as a basis for the integration. We linearly combine the affinities to fully retain the data manifold structure and intrinsic information, while largely reducing the computational complexity. Furthermore, our approach implements self-diffusion process by iteratively propagating the integrated network to improve the patients’ similarities for the downstream clustering analysis. To demonstrate the effectiveness of our MOSD, we carried out integrative subtypes identification across ten cancer types with three data types (Gene expression, DNA methylation, miRNA). Experiment results reveal that our MOSD approach outperforms the existing state-of-the-art integration methods in patients’ survival differences and running efficiency. Lastly, we comprehensively performed biological analysis of subtypes identified on colorectal and breast cancer.

Our key contributions in this work are summarized as follows: (1) MOSD uses local scaling method to construct patient’s affinity, which requires less hyper-parameters settings and can eliminate the scale differences. (2) Weight assignments derived from the features size relatively reflect the contribution of each data type to the integrated network. (3) Self-diffusion applied in MOSD has nearly no parameters tuning and can greatly enhance the clustering of integrated network, making it faster than most prior methods.

## Methods

Our MOSD approach includes three main steps: (1) Constructing local scaling affinity to measure patients’ similarities on multi-omics data. (2) Calculating the weight on each omics and linear combine the affinities into one network. (2) Performing self-diffusion process to enhance the patient-to-patient similarities learning. (3) Identifying cancer subtypes by clustering on the diffusion map and performing biological analysis.

### Local scaling affinity construction

Assuming we have ${\text{n}}$ samples (e.g., patients) and ${\text{m}}$ omics. Let $X_{m}$ denote the measurement,$X_{m}$ has dimensions $n \times d_{m}$, where $d_{m}$ is the number of features in measurement. Given a graph model $G{\mathbf{ = (}}\Omega ,\,{\mathbf{A)}}$, where $\Omega { = }\left\{ {x_{i} } \right\}_{i = 1}^{n}$ represents data samples, a $n \times n$ local scaling affinity matrix ${\mathbf{A}}(i,j) \in [0,1]$ is defined as follows [17]:

1 $$  {\mathbf{A}}(i,j) = \exp \left\{ {\frac{{ - d^{2} (x_{i} ,x_{j} )}}{{\sigma _{i} \sigma _{j} }}} \right\}  $$

where $d({x}_{i},{x}_{j})$ is Euclidean distance between ${x}_{i}$ and${x}_{j}$.The distance between ${x}_{i}$ and ${x}_{j}$ as “seen” by ${x}_{i}$ is $d({x}_{i},{x}_{j} )/{\sigma }_{i}$ while the converse is$d({x}_{j},{x}_{i} )/{\sigma }_{j}$. Local scale $\sigma_{i}$ can be obtained by calculating $d(x_{i} ,x_{k} )$,where $x_{k}$ is the $k^{\prime}$ neighbours of sample $x_{i}$. This affinity definition allows self-tuning of sample-to-sample distance, automatically keeping balance among multiple scales in data structure.

### Affinities integration

We define the weight coefficient $w$ for each data type as follows:

2 $$  p_{i}  = \exp \left( {\frac{{d_{i} }}{{\sum\limits_{m} {d_{m} } }}} \right)  $$

3 $$ w_{i} = \frac{{p_{i} }}{{\sum\limits_{m} {p_{m} } }} $$

Then, the integrated similarity matrix is calculated by: ${\mathbf{S}} = \sum\limits_{i = 1}^{m} {w_{i} } {\mathbf{A}}_{i}$.

### Performing self-diffusion

To enhance the real connections in network and facilitate the downstream clustering performance, we implement a self-diffusion process using the following step:

4 $$ {\mathbf{S}}^{t + 1} = \alpha {\mathbf{S}}^{t} {\mathbf{\rm T}} + (1 - \alpha ){\mathbf{I}}_{N} $$

where ${\mathbf{\rm T}}$ is the row-normalized transition matrix of ${\mathbf{S}}$ according to SNF and defined as:

5 $$ {\mathbf{\rm T}}(i,j) = \frac{{{\mathbf{S}}(i,j)}}{{\sum\nolimits_{k \in knn(i)} {{\mathbf{S}}(i,k)} }}\delta \{ j \in knn(i)\} $$

Self-diffusion process assigns similarities to non-neighbors relying on the assumption that local similarities in a network are more reliable than remotes ones.

### Network clustering

To obtain the labels of patients in cancer subtyping, we used spectral clustering to perform network clustering. Spectral clustering solves the graph optimization by minimizing RatioCut [18].

6 $$  \begin{array}{*{20}c}    {\underbrace {{\arg \min tr\left( {{\mathbf{H}}^{T} {\mathbf{LH}}} \right)}}_{{\mathbf{H}}}} & {s.t.{\mathbf{H}}^{T} {\mathbf{H}} = {\mathbf{I}}}  \\   \end{array}   $$

where ${\mathbf{L}}$ is the Laplacian matrix denoted by ${\mathbf{L}} = {\mathbf{I}} - {\mathbf{D}}^{ - 1/2} {\mathbf{SD}}^{ - 1/2}$.${\mathbf{D}}$ represents the graph degree matrix. By finding the minimum $k$(clusters) eigenvalues of ${\mathbf{L}}$, corresponding $k$ eigenvectors can be obtained. ${\mathbf{H}}$ is a $n \times k$ dimensional matrix formed by the $k$ eigenvectors. Compared to other clustering algorithms, spectral clustering which is based on graph cutting theory has more advantages in capturing graph structure.

### Estimating the optimal clustering number

We provide a separation cost method to estimate the optimal clustering number according to [17]. Given a set of clustering numbers $C$, Separation cost method aims to find an index matrix ${\mathbf{Z}}(R) = {\mathbf{XR}}$, ${\mathbf{X}} \in {\mathbb{R}}^{n \times C}$, ${\mathbf{R}} \in {\mathbb{R}}^{C \times C}$ to satisfy the following formula:

7 $$ [{\mathbf{M}}(R)]_{i} = \max_{j} [{\mathbf{Z}}(R)]_{i,j} $$

where ${\mathbf{X}}$ is the matrix composed by the eigenvectors of Laplacian matrix ${\mathbf{L}}$. ${\mathbf{R}}$ is its rotation matrix. The separation cost function is defined as follows:

8 $$ J(R) = \sum\nolimits_{i,j} {\frac{{\left[ {{\mathbf{Z}}(R)} \right]_{i,j}^{2} }}{{\left[ {{\mathbf{M}}(R)} \right]_{i}^{2} }}} $$

The optimal clustering number is the $C$ that minimizes the function $J(R)$.

### Statistical analyses

Statistical analyses are performed based on R (version 4.1.3, www.r-project.org). Kaplan–Meier method [19] is used to perform survival analyses and statistical significance is evaluated by log-rank test from ‘survival’ package [20]. Differential gene expression analysis is calculated among subtypes using ‘limma’ R package [21]. Gene set enrichment analysis (GSEA) is performed using ‘HTSanalyzeR’ package [22]. *P-*value of less than 0.05 is considered significant for all tests.

## Results

### Simulation evaluation of MOSD

**Fig. 2:**
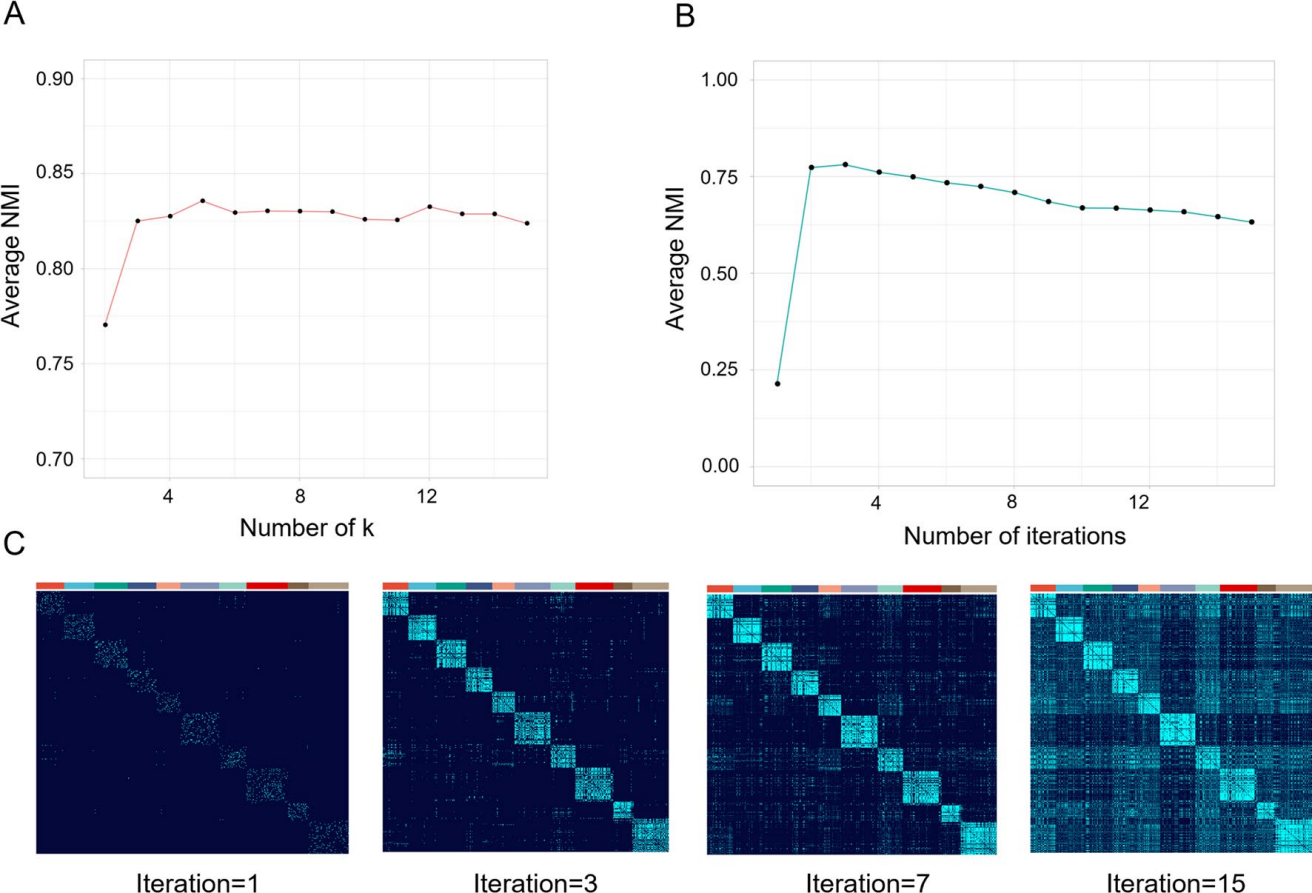
Simulation on evaluation study**.** Clustering performance, measured by averaged NMIs are shown for selecting the appropriate local scale neighbors(k = 5) (**A**) and self-diffusion iterations(t = 3) (**B**). **C** Cell-to-cell similarities matrices represented by different iterations (t = 1, 3, 7, 15). Clusters are arranged according to the ground-truth labels

To select the appropriate numbers of neighbors in affinity construction and the iterations in self-diffusion step, we conducted a simulation experiment to approximate the parameters. Since the truth labels of patients in cancer subtyping is unknown, we applied ‘Splatter’ R package [23] to simulate scRNA-seq read count data. 500 cells were simulated with 1000, 2000, 5000 genes forming ten groups, respectively. Three affinities were first created under the different gene features varying neighbors *k* from 2 to 15. MOSD integrated the three affinities into one final network, followed by self-diffusion process implemented on the network with iterations from 2 to 15. Spectral clustering was performed on the network to obtain the cell labels. We used normalized mutual information (NMI) [24] as a measurement of consistency between the ground truth and the obtained labels. The range value of NMI from 0 to 1, where a higher value yields higher concordance. We calculated the average NMI values under the different settings of neighbors and iterations. The results show that the average NMI reaches the highest value when neighbors *k* = 5(NMI = 0.76) (Fig. 2A) and iterations t = 3(NMI = 0.84) (Fig. 2B). To further explore the effectiveness of network denoising with self-diffusion, we visualized the integrated similarity matrix under iterations t = 1,3,7,15 with *k* = 5. Visual inspection of the diffused matrix reveals an enhancement within each cluster and clearer boundaries between clusters. It can be easily observed that the connectivity within clusters shows the most tightness when the iterations t = 3, while too many iterations in diffusion process may generate redundant information in the network (Fig. 2C).

To further confirm the selected parameters, we additionally conduct an experiment to learn parameters on muti-omics dataset of breast cancer(n = 628). We used log-rank p-value derived from survival analysis to approximate the parameters. Patient-to-patient affinities were constructed under three data levels (Gene expression, DNA methylation, miRNA) varying neighbors k from 2 to 15. The three affinities were integrated into one final network and self-diffusion process was then performed on the network with iterations from 2 to 15. Boxplot shows that k = 5 and t = 3 are the optimal parameters, indicating by the relatively significant log-rank p-value (Additional file 2: Fig S1). The optimal parameters are consistent with the result from single-cell simulation datasets.

### Performance evaluation and comparison of MOSD

We applied MOSD on ten cancer types including 2410 patients obtained from TCGA. The datasets are as follows: Adrenocortical carcinoma (ACC), Breast invasive carcinoma (BRCA), Colorectal adenocarcinoma (CRC), Glioblastoma multiforme (GBM), Glioma (GBMLGG), Head and Neck squamous cell carcinoma (HNSC), Kidney Chromophobe (KICH), Acute Myeloid Leukemia (LAML), Mesothelioma (MESO), Uveal Melanoma (UVM). For each cancer type, three omics are analyzed: Gene expression, DNA methylation, miRNA expression. The Detail information of datasets sizes and features can be found in Additional file 1: Table S1. We assessed the clustering performance based on three evaluation criteria: i. Differences in patient survival indicated by log rank test *p*-value. ii. Silhouette width (measuring cohesion and separation within clusters of data). iii. Computational efficiency (running time).

**Fig. 3:**
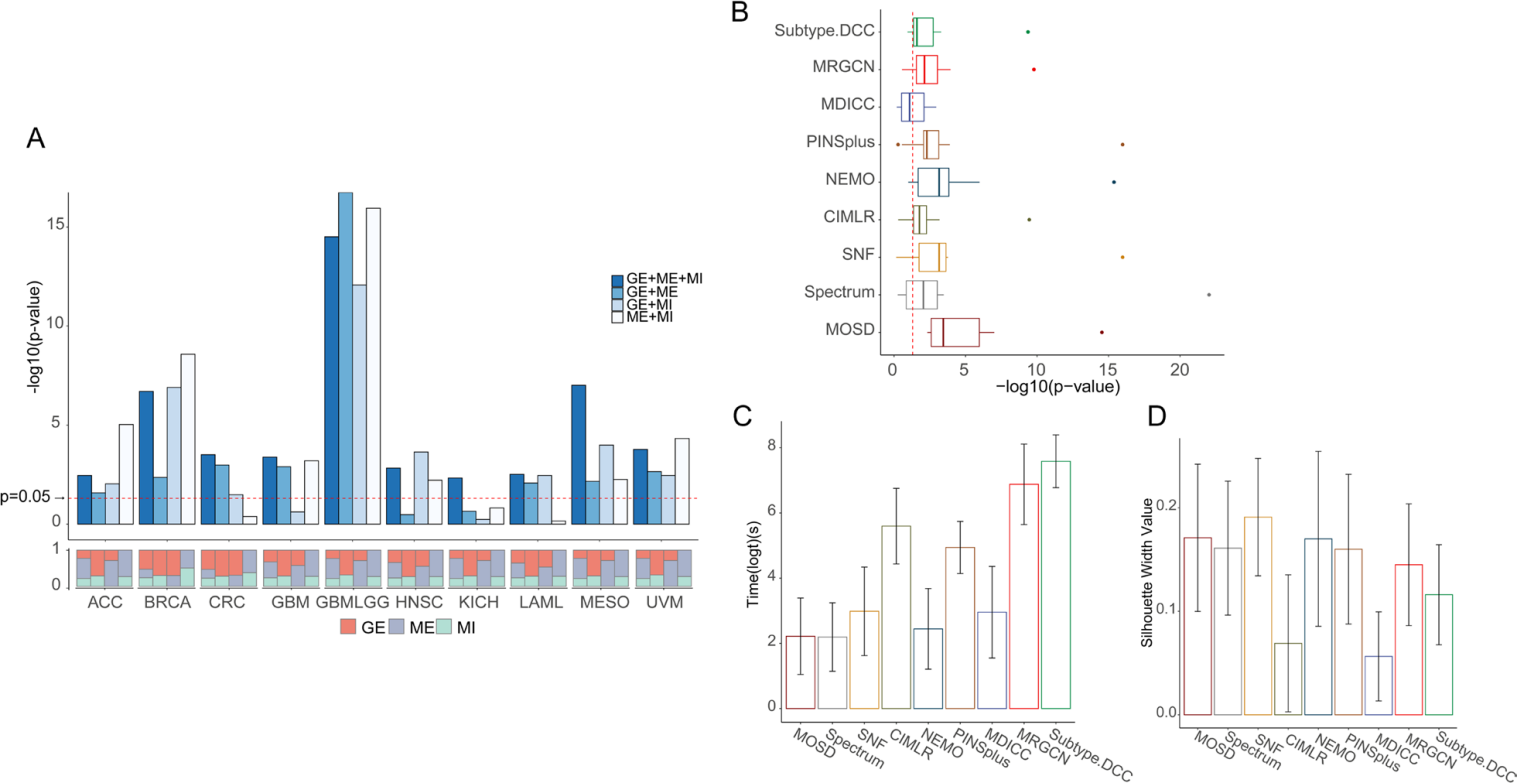
Survival analysis using combinations of different data types and benchmark experiment on ten cancer types. **A** Up: Survival differences comparison using different omics combination for can subtyping. Down: Weight values for each data type. **B** Boxplot shows the survival performance of the nine integration methods on ten cancer datasets. MOSD outperforms other eight methods indicated by the -log10(p-value). Meanwhile, our method exhibits impressive computational efficiency (**C**) and relatively better silhouette width (**D**) among the nine methods. Bars indicate the average performance over the ten cancer types. Error bars is the 95% confidence interval

First, to fully demonstrate the effectiveness of our MOSD, we explored the influence of combinations using different measurements on cancer subtyping. Three data measurements have four combination modes (Fig. 3A). We focused on the question that whether there are combinations of measurements that enable effective performance on all datasets. Affinity matrix for each data measurement was first created. The affinities were then assigned different weights to perform the integration, followed by self-diffusion process and spectral clustering on the integrated network. The optimal clustering numbers for the ten cancer types are estimated by separation cost method, indicated by the largest drop in the separation cost values (Additional file 2: Fig S2). Log-rank test p-value derived from survival analyses was used to evaluate the clinical relevance of clustering results. We find that different combinations of data measurements affect the subtypes identification for patients’ survival difference on the ten cancer types. An interesting observation is that the combination of three measurements (GE + ME + MI) reveals all very significant survival differences on the used cancer datasets.

To benchmark against other methods for integrative subtyping, we then performed comparisons of MOSD with eight other integration methods using the combination of three measurements on the ten cancer types. The 8 integration methods include Spectrum [11], SNF [6], CIMLR [9], NEMO [7] PINSplus [10], MDICC [25], MRGCN [26], and Subtype. DCC [27].Boxplot demonstrates that log rank p-value derived from MOSD reveals the significance across all the ten cancer types (Fig. 3B), outperforming other methods in survival analysis. The reason may be that the weight assignment for each data type is more biologically meaningful, resulting in more significant association between the identified subtypes and patient outcomes.

Extensively, to compare the weight assignment in MOSD, we also given equal weight on single-omic to examine the clustering effectiveness. Survival analysis indicates that the clinical association of the clustering results derived from the weight assignment in MOSD is more significant than that using equal weight on single-omic across the ten cancer types (Additional file 2: Fig S3).

We further quantitatively benchmark the computational efficiency and clustering performance on the ten cancer types. Remarkably, MOSD generally outperforms other benchmarked methods on the running time (Fig. 3C). Though Spectrum method runs slightly faster than MOSD, the survival differences in Spectrum is worse. Subtype.DCC approach is proved to be the most time-consuming since it requires deep neural network to learn clustering-friendly representations. The clusters cohesion can be measured by the silhouette width. In our study, to make fair comparison, for both MOSD and the benchmarked methods, we take as input the concatenated measurements in the original space to calculate the silhouette width. We find SNF has the highest silhouette width value followed by our MOSD and NEMO, while MDICC performing the worst (Fig. 3D). Error bars indicate the 95% confidence interval. In summary, we demonstrate that MOSD’s survival analysis archives comparative performance as some existing integration methods, while being relatively fast and efficient with fewer parameters tuning.

### Case study on colorectal cancer

**Fig. 4:**
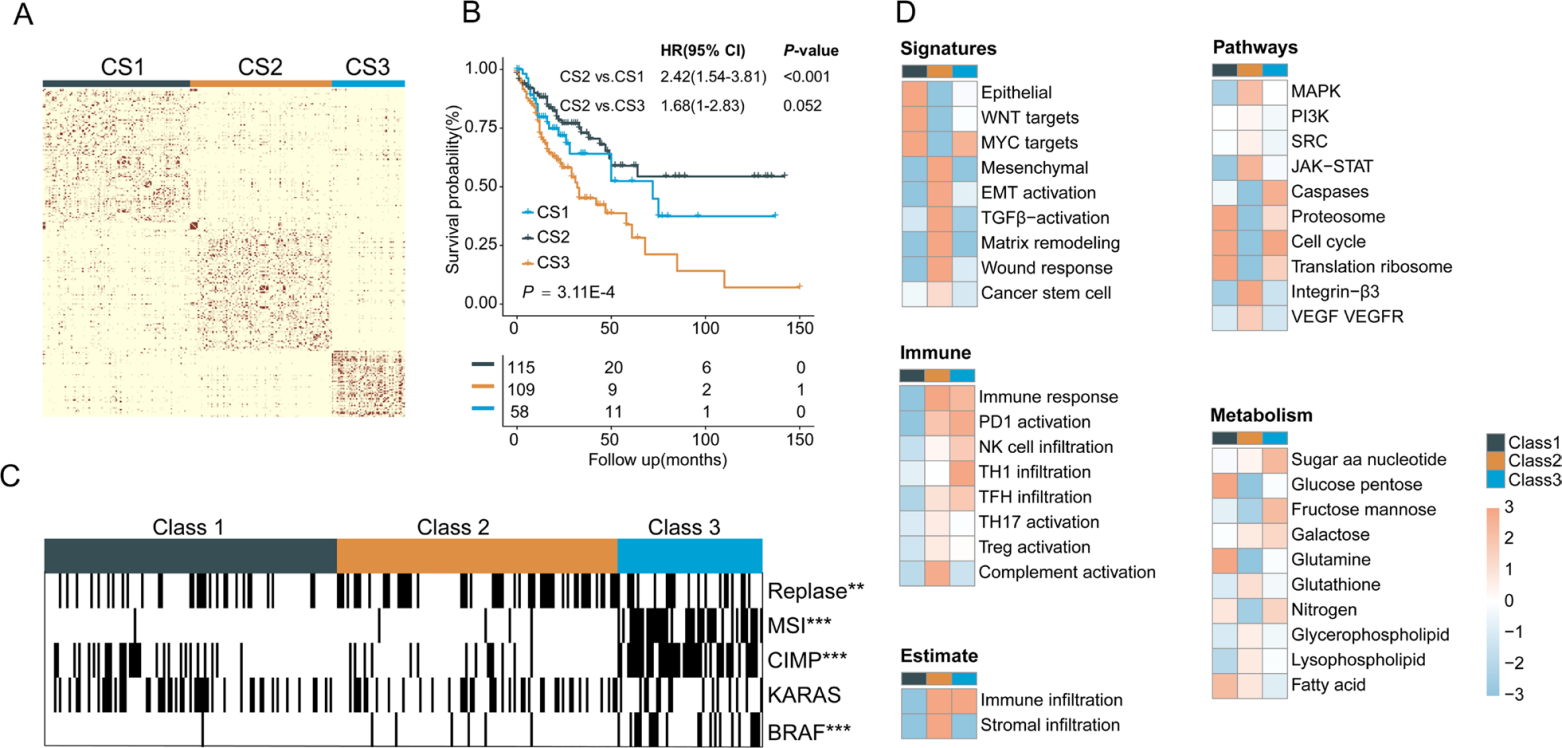
Case study on colorectal cancer. **A** Integrated similarity matrix for 297 patients with CRC. Clusters (CS1-CS3) are ordered by spectral clustering labels from integrated network and reveal relatively stronger inter-cluster similarity. **B** Kaplan–Meier curves of disease-free survival for the subtypes identified using MOSD (*p* = 3.11E-4, log-rank test). **C** The identified subtypes (CSs1-3) show significantly associated with some common mutations (*0.05; **0.01; ***0.001). **D** Gene set enrichment analysis using mRNA expression. The MOSD subtypes reflect different interests in some signatures or pathways

Colorectal cancer (CRC) is the third leading cause of cancer-related mortality worldwide. CRC has been proved be a heterogeneous disease with distinct molecular properties and is well suited to study the genomic subtyping. Tremendous effort has been dedicated to CRC subtyping, but only the gene expression is used [28, 29]. We validate the performance of MOSD using the three measurements of colorectal adenocarcinoma. Specifically, for better exhibiting the subtypes’ biological characteristics, we performed median absolute deviation (MAD > 0.5) to select the highly expressed features datasets. MOSD estimates 3 to be the optimal clustering number on the integrated matrix as it results in the largest drop in the value of separation cost (Additional file 2: Fig. S1). The patient-to-patient similarities for 297 patients were represented by similarity matrix (Fig. 4A). The relatively few edges between clusters illustrate the tightness of connectivity within clusters. To examine the clinical significance of MOSD CRC subtypes, clinical outcomes and characteristics are compared among the subtypes. The subtypes identified using MOSD show a significant association with disease free survival (Fig. 4B, P = 3.11E-3, log-rank test). Subtype CS3 is significant associated with mismatch repair (MSI) (*p* < 0.001) and shows a much higher CpG island methylator phenotype (CIMP) (*p* < 0.001) as well as BRAF-mutant (*p* < 0.001), indicating that it demarcates the well-characterized MSI/CIMP + subset of CRC (Fig. 4C). Univariate and multivariate Cox regression analyses were performed for CRC cancer to compare the MOSD subtypes with some clinical factors including Sex (Female vs. Male), TNM stage (III–IV vs. I–II), CIMP (High vs. Low), MSI (dMMR vs. pMMR), as well as MOSD subtypes (CS 2 vs CSs 1,3). MOSD subtypes are the most significant factors next to TNM stage in univariate and multivariate analysis (Table 1).

**Table 1:** Univariate and multivariate analyses for colorectal cancer

	Univariate analysis		Multivariate analysis	
	HR (95% CI)	p value	HR (95% CI)	p value
Sex (Female vs. Male)	0.98(0.66 – 1.45)	0.93	0.9 (0.6 – 1.35)	0.6
TNM stage (III–IV vs. I–II)	1.49 (1.21 – 1.83)	0.00014	1.45(1.18 – 1.79)	0.00044
CIMP (High vs. Low)	0.86 (0.55 – 1.34)	0.49	0.89 (0.54 – 1.46)	0.64
MSI (dMMR vs.pMMR)	0.98(0.58 – 1.67)	0.95	1(0.54 – 1.84)	0.99
MOSD (CS 2 vs CSs 1,3)	2.13 (1.44 – 1.3.15)	0.00017	1.91(1.26 – 2.89)	0.002

To further investigate the biological function characterized by each MOSD subtype, we performed differential gene expression analysis and identified subtypes-specific biological pathways based on gene set enrichment analysis (Fig. 4D). Subtype CS1 is enriched in epithelial differentiation and shows a significant upregulation of WNT and MYC, both of which are related to CRC carcinogenesis. Subtype CS2 is characterized by activated transforming growth factor (TGF)-β signaling, matrix remodeling pathways and displays upregulated genes associated with epithelial-mesenchymal transformation (EMT). Moreover, gene expression of subtype CS2 is consistent with stromal infiltration. Subtype CS3 tumors are featured by gene expression associated with diffuse immune infiltration, mainly consisting of TH1 and cytotoxic T cells, and strong activation of immune evasion pathways, which are distinguishing features of MSI CRC [30].

### Case study on breast cancer

**Fig. 5:**
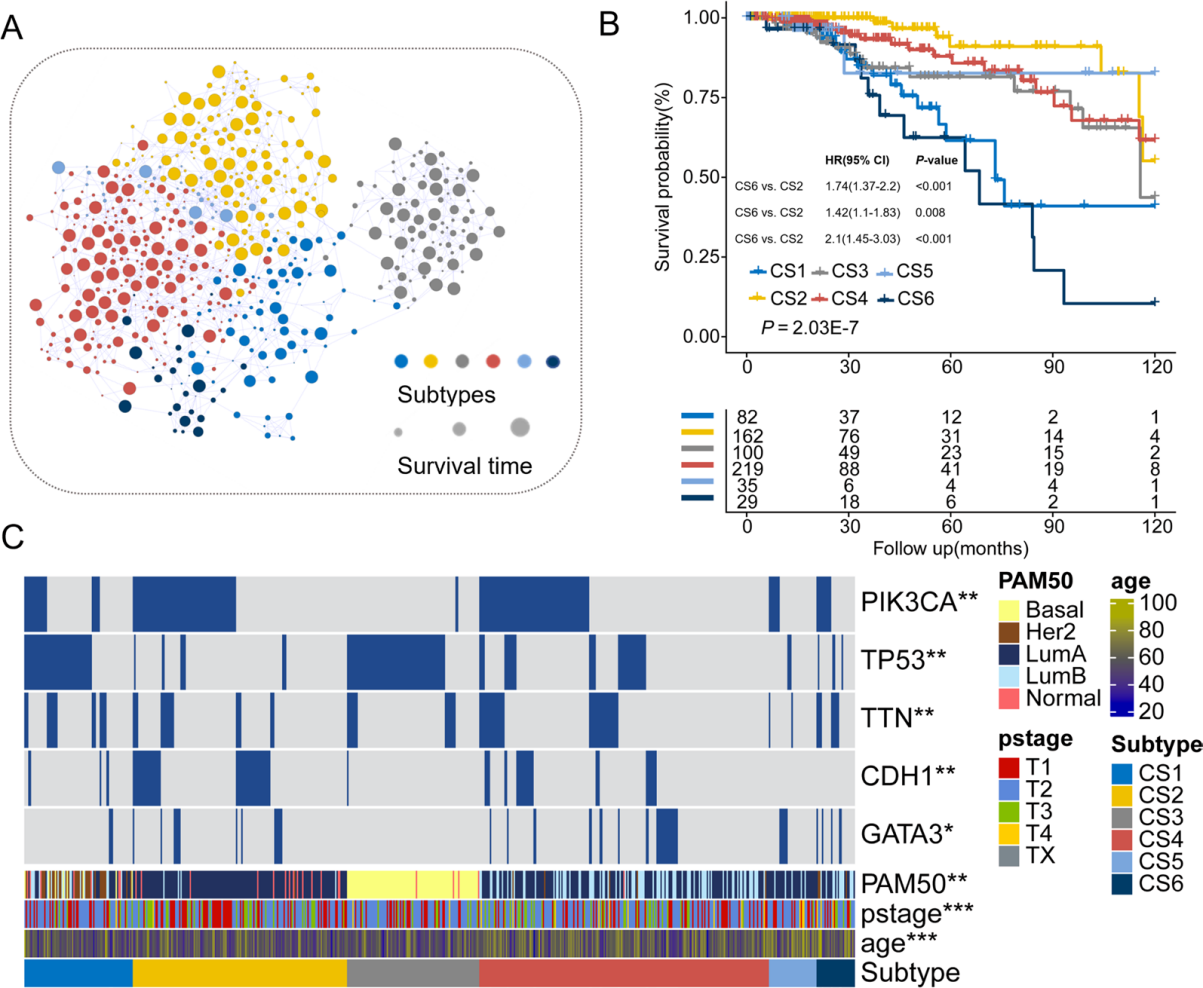
Case study on breast cancer. **A** Network similarity for 628 breast cancer patients, where nodes are patients and node size represent the length of survival time. **B** Kaplan–Meier plot for the six clusters identified by MOSD (*p* = 2.03E-7, log-rank test). **C** Frequency of some mutations in the six subtypes (*0.05; **0.01; ***0.001)

**Fig. 6:**
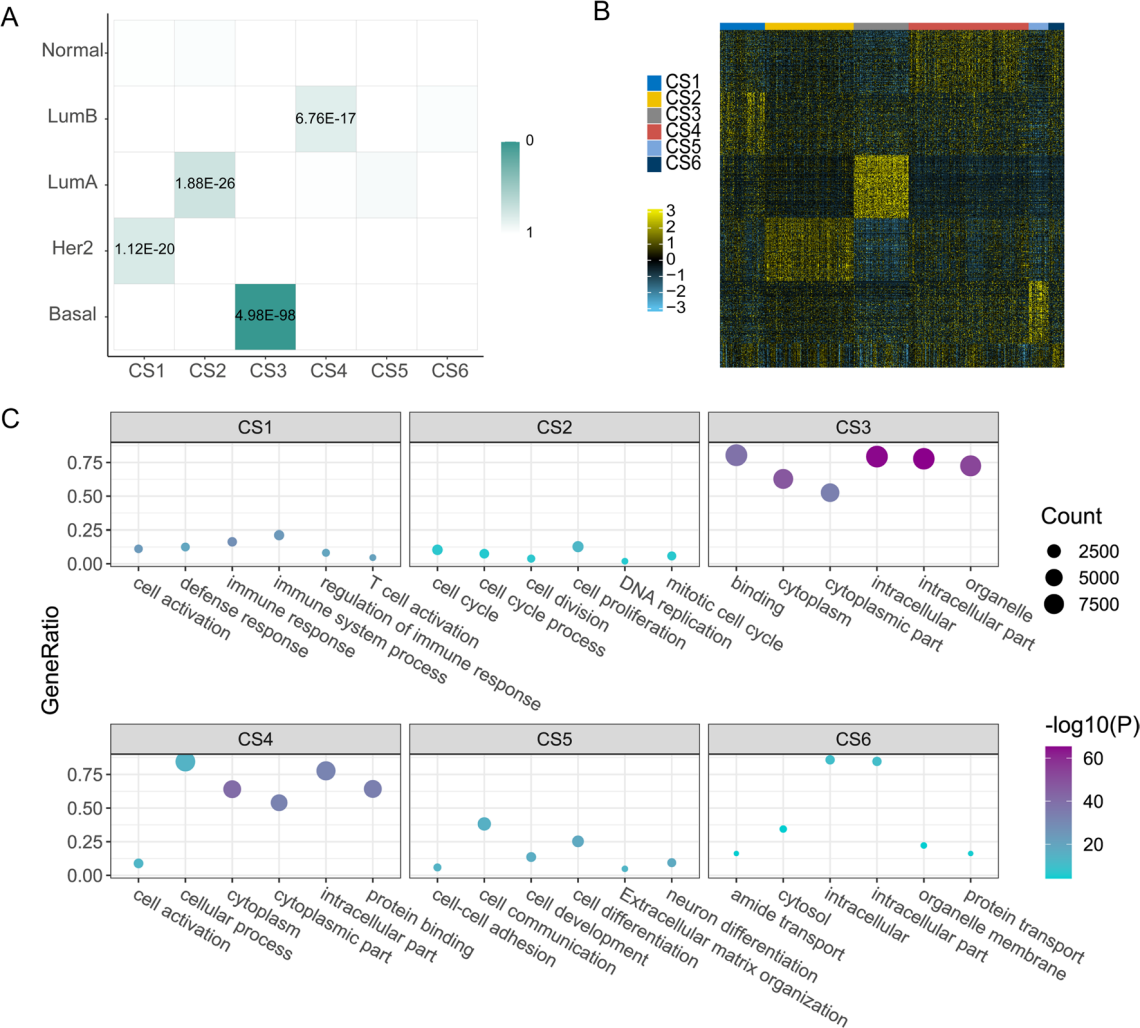
Biological analysis of MOSD breast subtypes. **A** Hypergeometric test of the MOSD subtypes between the known five breast subtypes. **B** Heatmap of top differentially expresses genes in the in the six subtypes. **C** Biological pathways reflected in the six subtypes using gene set enrichment analysis

Breast cancer is the world’s most primary causes of cancer death in women. Like other cancers, breast cancer is a highly heterogeneous disease underling different pathological characteristics and clinical response. Therefore, accurate understanding of subtypes’ biological function that are associated with clinical features has the vital significance on treatment decision. Based on gene expression profiling, breast cancer is identified into five molecular subtypes: Luminal A, Luminal B, HER-2 positive, basal-like and normal-like [31]. Here, we seek to investigate the MOSD’ ability to dissect the heterogeneity of breast cancer using the three measurements. MOSD classify 628 breast cancer patients into 6 clusters (CS1-CS6) on the integrated graph. We first explore the separation of the 6 clusters by patient network (Fig. 5A), where nodes represent patients and node size reflects survival time. The patient’s network is effective in visual representation and can emphasize the detail similarity patterns in integrated map. Patient samples classified to different subtypes are tightly distributed in the integrated network. To evaluate the clinical relevance of the six clusters, we performed survival analyses. The identified six subtypes based on MOSD are significantly associated with overall survival (Fig. 5B, P = 2.03E-7, log-rank test) and subtype CS6 shows the worst overall than the rest of clusters. To further characterize the identified clusters, mutational frequency is then calculated among the six clusters. We determine the frequency of PIK3CA, TP53, TTN, CDH1, GATA3 mutations in all patients (Fig. 5C). Subtype CS3 reveals a striking association with TP53 mutation which acts as a tumor suppressor and regulates cell division. PIK3CA mutation is reported highly represented in ER + /HER2- breast cancer [32]. The results shows that PIK3CA mutation is enriched in subtypes CS2 and CS4, of which is significantly associated with Luminal A and Luminal B subtypes, respectively. To quantitively reveal the association between MOSD subtypes and PAM50 breast subtypes, we performed the hypergeometric test (Fig. 6A). Based on the calculation, subtype CS1, CS2, CS3, CS4 recapitulated the ‘Her2’, ‘LumA’, ‘Basal’ and ‘LumB’ subtypes previously reported. Univariate and multivariate analyses show that MOSD subtypes are the significant factors of survival as well as other clinical characteristics like age, TNM stage and chemotherapy (Table 2). To examine biological characterizations of the six clusters, we analyzed differentially expressed genes in each cluster as compared to the others (Fig. 6B). Meanwhile, we performed gene set enrichment analysis to identify the top statistically significant pathways in each cluster (Fig. 6C). GSEA results show that subtype CS1 is related to some immune pathways. Subtype CS2 which has good prognosis is featured by activated insulin-like growth factor and extracellular matrix (ECM). This subtype is the most common subtype in breast cancer. Subtype CS3 compatible with Basal-like subtype has high histological grade and is implicated in facilitating cellular proliferation and cell migration.

**Table 2:** Univariate and multivariate analyses for breast cancer

	Univariate analysis		Multivariate analysis	
	HR (95% CI)	p value	HR (95% CI)	p value
Age (> = 65 vs. < 65)	2.72 (1.72 – 4.29)	< 0.0001	1.97 (1.17 – 3.32)	0.01
Chemotherapy (0 vs. 1)	1.77 (1.34 – 2.34)	< 0.0001	1.61 (1.21 – 2.26)	0.0017
TNM stage (III–IV vs. I–II)	2.02 (1.25 – 3.26)	0.004	2.67 (1.61 – 4.42)	0.0002
Tumor location(R vs. L)	0.75 (0.47 – 1.19)	0.22	0.61 (0.37 – 0.99)	0.048
MOSD (CS 6 vs CSs 1–5)	1.26 (1.14 – 1.40)	< 0.0001	1.32 (1.16 – 1.5)	0.0023

## Discussion

Cancer is one of the most heterogeneous diseases, underlying multiple subtypes with distinct molecular properties and diverse morphological profiles.

Accurately identifying cancer subtypes is essential to unfold molecular features and correlate them with patients’ outcome. Most studies for cancer molecular subtyping are mainly based on unsupervised clustering of single-omic data [33, 34], particularly gene expression profiling. But cancer is a phenotype that accumulates at multiple levels of the biological system, from the genome to the proteome. Integrating multi-omics data can facilitate the understanding of potential biological mechanisms and improve clinical outcomes [35, 36]. In the paper, an efficient multi-omics integration framework, namely MOSD is proposed for cancer subtypes identification. Different from many existing multi-omics integration methods, MOSD aims at constructing patient-to-patient network to preserve omics expression and similarity relationships by creating local scaling affinity which infers the self-tuning of samples distances. Also, MOSD try to deal with the tackle of weight assignment in each data type. The fractionation of data features size is utilized to assign a weight in each omic with the assumption that larger features contain more heterogeneous information and contribute more to the final integrated graph. Based on the integrated network, self-diffusion process is applied to enhance the similarity learning. Self-diffusion implements a dynamic diffusion process that uses local graph structures to denoise the networks, while need few parameters tuning. The important advantage of the self-diffusion is that the weak similarities disappear to reduce feature redundancy and strong similarities are preserved along the graph diffusion, largely facilitating the downstream clustering efficiency with biological analysis.

We extensively evaluate the effectiveness of MOSD across ten cancer types. MOSD successfully stratify patients into clinically relevant subtypes with significantly different survival rates on the used datasets. The reason relies on that MOSD can learn appropriate patient-to-patient distances that reveal the similarity structure to reduce noise and reductant information and the weight assignment is biologically suitable to uncover the patient outcome associated with clusters. Benchmark experiment shows that MOSD outperforms eight other state-of-art integration methods in survival differences of discovered subtypes and shows better computational complexity. Finally, we perform comprehensive biological analysis of subtypes identified on colorectal and breast cancer. MOSD is an open data integration framework not only in cancer subtyping but also can be easily utilized in more omics scenarios. However, our MOSD has some limitations. MOSD cannot handle the partial data since the integrated step need the same size of dimension in affinities.

## Conclusion

Stratifying cancer patients into molecular subtypes is important to understand intratumor heterogeneity with malignant progression. Integrated analysis of multi-omics datasets provides opportunity to discover such tumor subclones. MOSD’ simplicity, robustness, efficiency make it an ideal approach to identify subtypes that reflect intratumor heterogeneity and can be extended to further use in clinical practice. We believe that machine leaning based cancer subtyping strategies will continually contribute more to patients’ personalized treatment and understand the caner biology.

### Supplementary Information

**Additional file 1: Table S1. **A summary of datasets used in this study.

**Additional file 2: Fig. S1. **Experiment on muti-omics dataset of breast cancer shows the optimal parameters are k=5 (**A**) and t=3 (**B**). **Fig. S2. **The optimal clustering numbers for the ten cancer types are estimated by separation cost method. **Fig. S3. **Comparison of weight assignment in MOSD approach.

## Data Availability

Publicly available data were analyzed in this work. ACC, GBM, GBMLGG, HNSC, KICH, LAML, MESO, UVM datasets were obtained from Nguyen et.al [10]. BRCA, CRC datasets were downloaded from The Cancer Genome Atlas (TCGA) (https://www.cancer.gov/ccg/research/genome-sequencing/tcga).
