# Supplementary material for: Multi-omics integration with weighted affinity and self-diffusion applied for cancer subtypes identification
Source: J Transl Med. 2024 Jan 19;22:79. doi: 10.1186/s12967-024-04864-x (PMC10799401; doi:10.1186/s12967-024-04864-x)
Supplement: Supplementary file 1 — Additional file 1: Table S1. A summary of datasets used in this study. [file 12967_2024_4864_MOESM1_ESM.docx]

**Table S1.** A summary of datasets used in this study

|  | **ACC** | **BRCA** | **CRC** | **GBM** | **GBMLGG** | **HNSC** | **KICH** | **LAML** | **MESO** | **UVM** |
| --- | --- | --- | --- | --- | --- | --- | --- | --- | --- | --- |
| **Number of samples** | 79 | 628 | 297 | 273 | 510 | 228 | 65 | 164 | 86 | 80 |
| **mRNA** | 20531 | 13029 | 20531 | 12042 | 20531 | 239886 | 20531 | 16818 | 20531 | 20531 |
| **DNA methylation** | 394014 | 1000 | 2080 | 22833 | 364743 | 375044 | 391298 | 22288 | 392708 | 394475 |
| **miRNA** | 1046 | 1046 | 705 | 534 | 2588 | 1046 | 1046 | 552 | 1046 | 2588 |
