# Supplementary material for: Multi-omics integration with weighted affinity and self-diffusion applied for cancer subtypes identification
Source: J Transl Med. 2024 Jan 19;22:79. doi: 10.1186/s12967-024-04864-x (PMC10799401; doi:10.1186/s12967-024-04864-x)
Supplement: Supplementary file 2 — Additional file 2: Fig. S1. Experiment on muti-omics dataset of breast cancer shows the optimal parameters are k=5 (A) and t=3 (B). Fig. S2. The optimal clustering numbers for the ten cancer types are estimated by separation cost method. Fig. S3. Comparison of weight assignment in MOSD approach. [file 12967_2024_4864_MOESM2_ESM.docx]

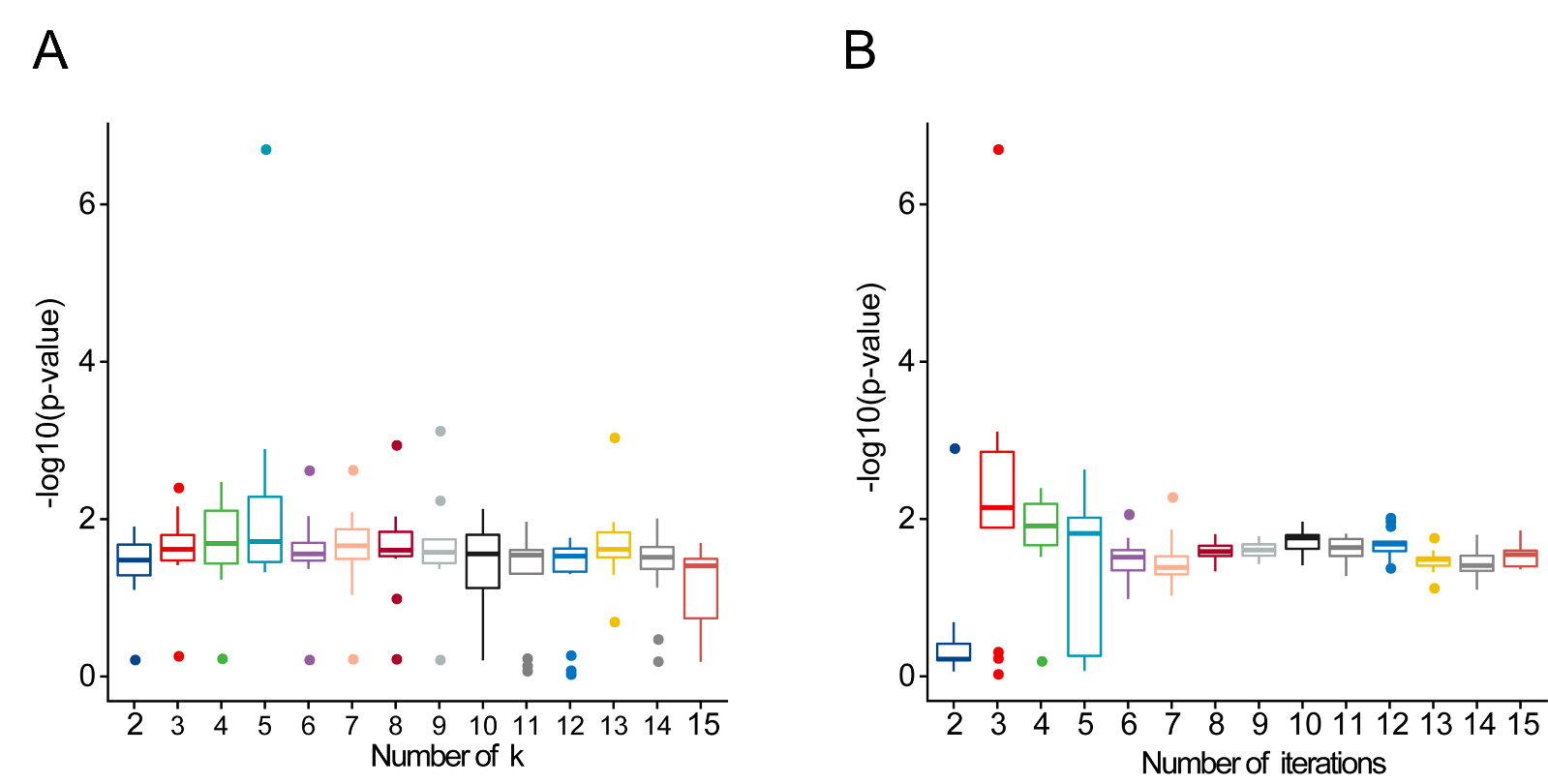


**Figure S1:** Experiment on muti-omics dataset of breast cancer shows the optimal parameters are k=5(A) and t=3(B).


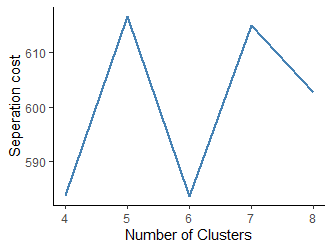

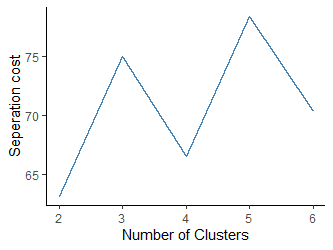


ACC

BRCA


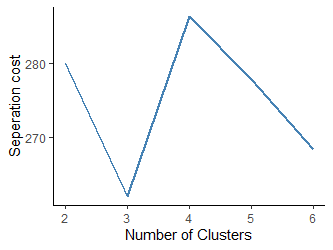


CRC


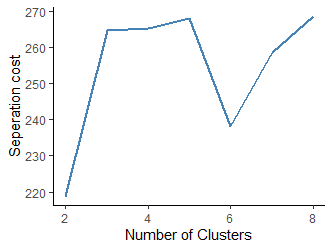


GBM


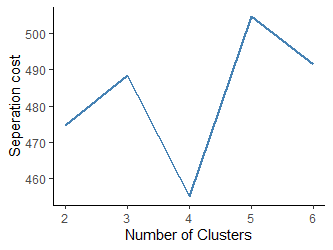


GBMLGG


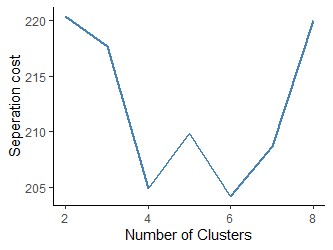


HNSC

KICH

LAML


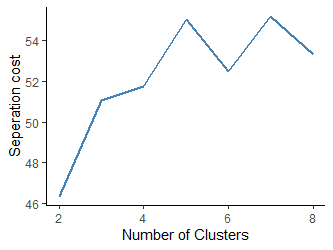

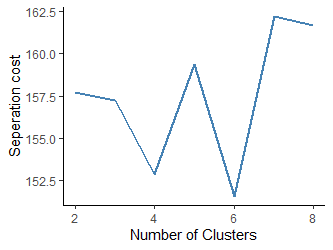


UVM

MESO


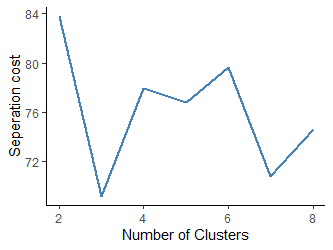

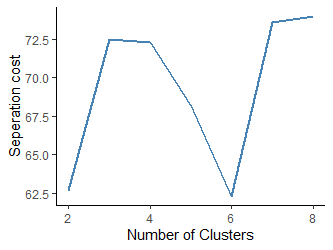


**Figure S2:** The optimal clustering numbers for the ten cancer types are estimated by separation cost method.


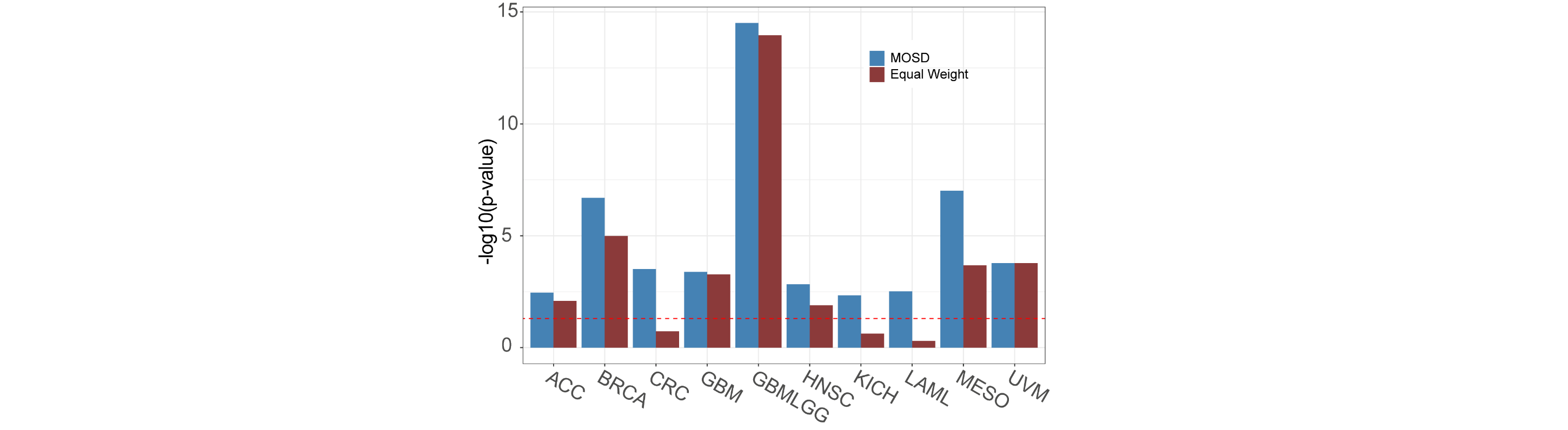


**Figure S3:** Comparison of weight assignment in MOSD approach.
